# Therapeutic Use of Integrin Signaling in Melanoma Cells: Physical Link with the Extracellular Matrix (ECM)

**DOI:** 10.3390/cancers17183037

**Published:** 2025-09-17

**Authors:** Katarzyna Adamiak-Nikolouzou, Andrzej T. Słomiński, Zofia Skalska, Iwona Inkielewicz-Stępniak

**Affiliations:** 1 Department of Pharmaceutical Pathophysiology, Faculty of Pharmacy, Medical University of Gdansk, Dębinki 7 Street, 80-211 Gdansk, Poland; 2 Department of Dermatology, Comprehensive Cancer Center, University of Alabama at Birmingham, Birmingham, AL 35294, USA; aslominski@uabmc.edu; 3 Pathology and Laboratory Medicine Service, VA Medical Center, Birmingham, AL 35294, USA

**Keywords:** cancer, melanoma, integrins, adhesion molecules, extracellular matrix

## Abstract

Extracellular matrix (ECM) macromolecules play a vital role in the regulation of cellular phenotype. Ongoing interactions of the extracellular matrix and cells via surface receptors can affect the cellular behavior selectively or non-selectively. Under physiological conditions, the ECM-cell interactions are essential for ensuring balance, whereas the dysregulation of these interactions can lead to the onset of diseases. Extensive knowledge of the integrins with two non-covalently linked α and β subunits plays a vital role in cell–cell adhesion and ECM interactions. The identification of a key adhesion signaling pathway may lead to new therapeutic strategies targeting melanoma cells. This review highlights the potential role of integrins as a selective target in melanoma therapy, which would reduce side effects and increase the effects of the treatment.

## Simple Summary

Understanding how the extracellular matrix (ECM)—the network surrounding cells—regulates cell behavior through integrins is essential for developing innovative strategies to treat melanoma. Investigating the role of integrins in controlling melanoma behavior can help identify new approaches to precisely target tumor cells. This review highlights recent advances in translational research between 2021 and 2025, focusing on potential melanoma therapies based on integrin signaling. These developments may pave the way for more selective and efficient treatment of melanoma patients.

## 1. Introduction

Melanoma, an aggressive skin tumor of melanocytic origin, significantly challenges public health due to its aggressive behavior, resistance to therapy of advanced disease, and complex biology [1]. Understanding its epidemiology, aggressive nature, and barriers to treatment is crucial for better patient outcomes. 

The global incidence of melanoma continues to rise. Data from the Rochester Epidemiology Project, spanning over 50 years, demonstrate evolving patterns in the rates of primary cutaneous melanoma [1,2]. While skin exposure to UV radiation (UVR), both from the sun and tanning beds, is the biggest risk factor for the development of cutaneous melanoma [3,4], many other environmental and genetic risk factors are now recognized as the cause of this disease [1]. It must be noted that the role of UVR, especially of its UVB spectrum, is complex and context-dependent [5,6,7]. The constitutional factors affecting susceptibility to melanoma development include low eumelanin or relatively high pheomelanin content in the skin [8,9,10], the presence of moles on the skin [11], genetic predisposition [12], previous history or family history of melanoma [13], and an immunosuppressive environment [14]. Modern medicine recognizes development as a complex process, shaped by genetic predisposition, environmental exposures, and lifestyle choices to varying degrees [1,7,15].

The rapid progression of melanoma and metastases to lymph nodes and distant organs, such as lungs [16,17], brain [18], and liver [19], significantly worsens the prognosis and highlights the critical need for early detection and potent systemic therapies [20]. Notably, advanced melanomas not only can autoregulate their tissue environment but also body homeostasis through production of diverse bioregulatory molecules [21]. The key to understanding and controlling metastasis in melanoma is consideration for the lining (stroma) and the level of oxidative stress in tumor cells. Rich lipid density in the stroma promotes high oxidative phosphorylation (OXPHOS). High oxidative phosphorylation (OXPHOS) in melanoma means that the tumor cells are heavily reliant on using oxygen and the mitochondrial electron transport chain to generate energy [22]. Therefore, metabolic balance and oxidative stress in melanoma cells, dependent on the environment, determine the ability to metastasize [23]. It is also dependent on the melanogenic activity [10,24,25].

**Figure 1:**
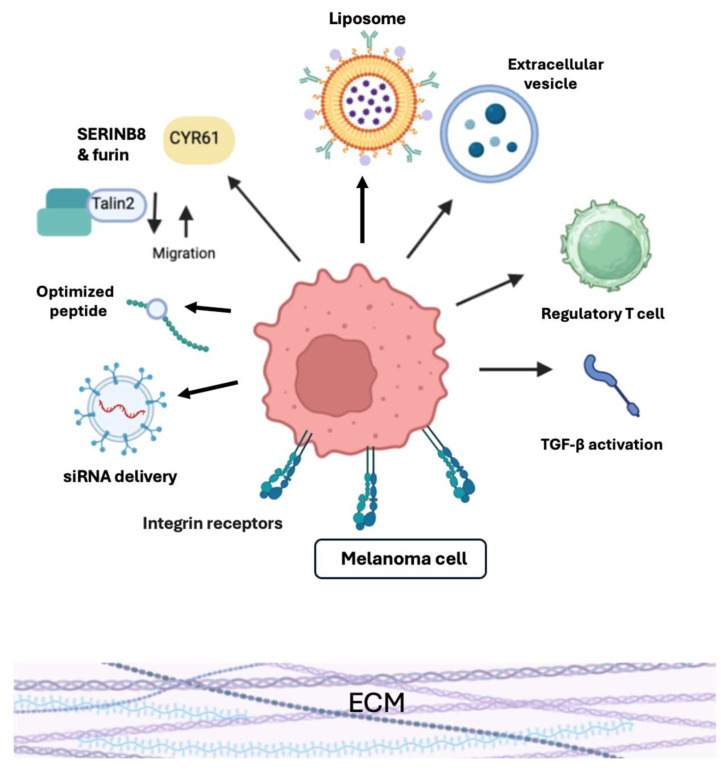
Emerging Therapeutic Approaches to Melanoma. Created with BioRender.com.

Despite recent progress, melanoma treatment, especially for advanced and metastatic forms, remains challenging. A major challenge is disease heterogeneity, where different melanoma cells react distinctly to therapies, often leading to drug resistance. These can be secondary to pathological and uncontrolled melanogenesis, generating an immunosuppressive and mutagenic environment within the tumor [10,24,26,27]. For example, while BRAF inhibitors revolutionized treatment for BRAF-mutated melanoma, resistance frequently occurs, limiting long-term efficacy [28]. Similarly, despite the impact of immunotherapy (e.g., immune checkpoint inhibitors), not all patients respond, and many of them develop primary or acquired resistance [29]. Reducing side effects of systemic therapies, like immune-related adverse events, requires careful clinical expertise [30]. The complexity of selecting therapies shown on Figure 1 and finding optimal patient-specific combinations remains an active research area and clinical challenge [1].

## 2. Integrin–Extracellular Matrix Interactions

### 2.1. The Role of Integrins

Integrins are a key family of cell surface receptors that mediate critical interactions between cells and their extracellular environment. Their unique biology is fundamental to various physiological and pathological processes, including oncogenesis and tumor progression, especially in melanoma [31].

Integrins are heterodimeric transmembrane glycoproteins, composed of one α (alpha) subunit and one β (beta) subunit. The human body has 18 different α and 8 β subunits. Their combinations allow the formation of 24 unique combinations of proteins, each exhibiting unique binding specificities and functional roles. These receptors link the extracellular matrix (ECM) to the actin cytoskeleton inside the cell, providing a mechanical bridge that allows cells to sense and respond to their physical surroundings [32]. Beyond this structural role, integrins play a key role in transducing signals bi-directionally across the cell membrane, affecting cell behavior [33].

Integrins play a key role in cell adhesion, enabling stable yet dynamic adhesion connecting cells to ECM components.

The adhesion is not static but dynamic, allowing cells to engage and disengage as needed for processes like migration. Beyond mechanical roles, integrins are powerful signaling agents. They mediate important signaling pathways that control various cellular functions, including cell survival, proliferation, and differentiation [34]. In a pathological context, such as cancer, these signaling functions contribute to the metastatic cascade. In cell migration, integrins regulate cell–substrate attachments, allowing cells to move across surfaces. This process involves cycles of integrin activation, binding to ECM, and later detachment, coordinated with cytoskeletal rearrangements. By translating extracellular cues into intracellular signals, integrins promote communication between the cell and its microenvironment, ultimately influencing fundamental cellular decisions [35].

### 2.2. Extracellular Matrix Remodeling in Melanoma

In melanoma, the expression and function of integrins are significantly altered compared to normal melanocytes, playing a crucial role in tumor progression and metastasis [36]. Melanoma cells frequently express specific integrin subtypes and promote their aggressive behavior. These integrins primarily mediate cell-ECM interaction and, to a lesser extent, intercellular adhesion. Based on their ligand specificity, integrins can be classified into collagen-binding (α1β1, α2β1, α10β1, and α11β1), laminin-binding (α3β1, α6β1, α7β1, and α6β4), leukocyte-associated integrins (αLβ2, αMβ2, αXβ2, αDβ2, αEβ7, and α4β7), and those that recognize the RGD (Arg-Gly-Asp) amino acid motif (α5β1, α8β1, αvβ1, αvβ3, αvβ5, αvβ6, αvβ8, and αIIbβ3) [37,38,39,40]. This broad diversity in integrin profiles suggests that each patient’s melanoma may exhibit a unique integrin signature.

**Figure 2:**
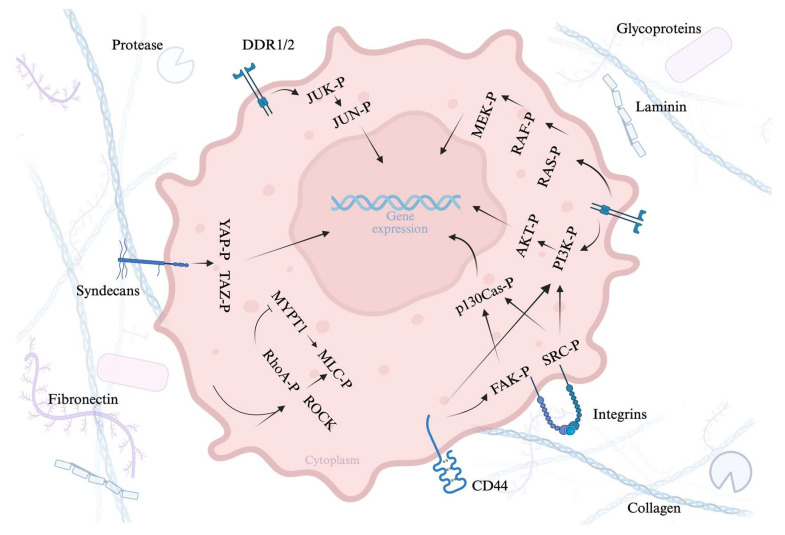
Cell–ECM interactions and downstream signaling pathways.

Figure 2 illustrates how extracellular matrix (ECM) components—like collagen, fibronectin, and laminin—interact with cell surface receptors (e.g., integrins, syndecans, DDR1/2, and CD44), triggering intracellular signaling pathways (e.g., PI3K/AKT, MAPK, Rho/ROCK, and YAP/TAZ). These pathways lead to changes in gene expression, ultimately affecting cell behavior and function.

The extracellular matrix (ECM) also contains hydrated, gel-forming macromolecules such as hyaluronan and proteoglycans alongside integrins that transmit adhesion signals, and various other components [41,42]. Understanding the ECM characteristics of a benign melanocytic nevus provides an essential baseline for investigating the pathophysiological remodeling that occurs in melanoma progression. In melanoma, the ECM undergoes significant remodeling compared to healthy tissue. Key differences include marked membrane disruption; modified distribution and accumulation of collagen IV and laminin around large cell aggregations; and a dense surrounding of melanoma nests by dermal collagens I, III, and VI. Additionally, levels of tenascin and fibronectin are significantly elevated throughout the dermis. This modified ECM forms a highly supportive matrix that actively promotes tumor growth and invasion [43,44].

### 2.3. Role in Tumor Progression and Metastasis

The “inside-out” signaling happens when a signal from inside the cell activates the integrin, even without an external ligand attaching. The “outside-in” signaling happens when a ligand attaches to the integrin from outside the cell. This causes the integrin to change shape, increasing its ability to bind to the ligand and leading to integrin clustering, which initiates cell adhesion to the extracellular matrix [45,46]. In melanoma, altered ECM composition directly impairs integrin signaling, disrupting normal cell communication. Disregulation contributes to more aggressive cellular behavior, including increased proliferation, motility, and resistance to therapy [47].

Integrins are key players in melanoma tumor progression and metastasis due to their modified expression in tumor cells. They also play a major role in the metastasis cascade, affecting fundamental processes such as cell adhesion, signaling, epithelial–mesenchymal transition (EMT), and the formation of new blood vessels (angiogenesis) [48]. Certain integrin subtypes can even direct melanoma cells to specific organs, promoting specific metastasis. As such, integrin-ECM signaling is a fundamental force behind the aggressive spread of melanoma [49].

### 2.4. Integrins and Therapy Resistance

Integrin-ECM signaling plays a pivotal role in therapeutic resistance, enabling tumor cells to survive under the pressure of treatment. In melanoma with BRAF mutations, resistance to targeted therapy, including dual treatment with MAPK and PI3K/AKT inhibitors, is a common challenge despite early efficacy [50].

Studies have shown that this resistance is mediated by ECM integrins α3β1 and α11β1. Once connected to the ECM, integrins activate Src kinase, a main mechanism driving resistance. The blockage of α3β1 and α11β1 integrins significantly inhibits the proliferation of resistant melanoma cells [51,52]. Due to their critical role in resistance mechanisms, integrins on melanoma cells can be targeted for selective treatment. This can be achieved through various strategies, such as blocking integrin–ligand interactions, inhibiting specific signaling pathways, or integrin-targeted therapies (e.g., antibodies or peptides) [53]. This work investigates why integrins are crucial in melanoma and highlights the role of the tumor microenvironment.

By examining their role in tumor growth, spread, and resistance to therapy, this research aims to understand how targeting integrin–extracellular matrix signaling can lead to new treatments.

## 3. Current Translational Research on Integrin Signaling in Melanoma Treatment

Integrins as transmembrane receptors play an essential role in cell migration, adhesion, and signaling in cancer growth [54,55,56,57,58,59,60,61,62,63,64,65,66]. In recent years, translational research has increasingly focused on integrin signaling pathways as potential therapeutic targets, especially in malignant melanoma, a highly aggressive form of skin cancer resistant to conventional therapies.

Translational research in the years 2021–2025 led to the potential integrin-targeted treatments and biomarkers [67,68,69,70,71,72,73,74,75,76,77,78,79,80].

Table 1 summarizes advances on integrin signaling in melanoma treatment in the past five years (2021–2025) based on in vivo models, whereas Table 2 presents in vitro studies. Table 3 presents research performed in both levels of evidence.

**Table 1:** In vitro studies on integrin signaling in melanoma treatment (2021–2025).

Year	Study Model	Key Findings	Mechanism of Intervention	Reference
2025	2D model/A 375, G-361,MeWo, MM127, MM370, RPMI-7951, SH-4, SK-MEL-1, A431, MCC13	The expression of integrin α1 (ITGA1) was elevated at both the mRNA and protein levels in drug-treated melanoma cells	ITGA1 may serve as a senescent melanoma cell biomarker	[81]
2024	2D model/ WM115, WM266-4	Melanoma-derived ectosomes are mediated by αvβ5 integrin	αvβ5 integrin present in melanoma ectosomes is a key driver of tumor-induced angiogenesis and may serve as a more effective target	[82]
2024	2D model/ SK-MEL-2, HEMn-MP	CYR61 increases melanoma cell proliferation and survival rate	Blockage of CYR61–integrin β3 may serve as a potential therapeutic target	[83]
2023	2D model/ A375, 1205Lu	SERPINB8 and furin regulate the expression of ITGAX, which promotes the proliferation of melanoma	ITGAX Knockout and SERPINB8 both inhibited the proliferation and invasion of melanoma cells	[84]
2023	2D model/ MDA- MB-435S	KANK2 interacts with talin2, resulting in increased sensitivity to PTX	The talin2 knockdown led to increased sensitivity to PTX and also reduced migration	[85]
2023	2D model/ A375P, K029A, SKMEL3	Possible targeted therapy for BRAF inhibitor–resistant melanoma characterized by epigenetically repressed PGC1α	Statin treatment blocks cell growth by lowering RAB6B and RAB27A prenylation and affects integrin localization and downstream signaling required for melanoma cell growth	[86]
2022	2D model/ SK-Mel-147	Knockdown of integrin α3β1 in SK-Mel-147 human melanoma cells led to a marked increase in cellular senescence of melanoma cells	Integrin α3β1 plays a protective role against cellular senescence in melanoma cells	[87]
2022	2D model/ 16F10	Silencing of STAT3 after treatment with anti-STAT3 siRNA-loaded liposomes triggers apoptotic activity in B16F10 melanoma cancer cells. C-RGD peptide targeted liposomal siRNA delivery system was able to induce apoptosis in a greater amount than non-targeted liposomes on overexpressed integrin αvβ3 receptor cells	C-RGD peptide targeted liposomal siRNA delivery system was able to induce apoptosis more than non-targeted liposomes on overexpressed integrin αvβ3 receptor cells	[88]
2022	2D model/ A375	NECTIN1 loss activates integrin-dependent matrix adhesion	Knockdown of integrins: β3, β4 and β5 results in reduced migration of NECTIN1-deficient cells	[89]
2021	2D model/ SK-Mel-147	Integrin α2β1 helps prevent senescence in SK-Mel-147 melanoma cells	The silencing of integrin α2β1 reduced cell proliferation and enhanced the percentage of SA-β-Gal-positive cells, a phenotypic feature of cellular senescence	[90]
2021	2D model/ WM793	ILK knockdown by siRNA suppresses melanoma cell growth by inducing autophagy through AMPK activation, and simultaneously initiates apoptosis	Combinatorial treatment of melanoma cells with CQ and siILK has a stronger antitumor effect than monotherapy	[91]
2021	2D model/ C32, SK-MEL-28	CD36 facilitates melanoma cell adhesion to the extracellular matrix (ECM)	CD36 as a regulator of VM by melanoma cancer cells that is facilitated via integrin-α3	[92]
2021	3D mouse model/ B16	Regulatory T (Treg) cells expressing the β8 chain of αvβ8 integrin (Itgβ8)—the main cell type in the tumors	Targeting the integrin αvβ8–TGF-β activation axis on Tregs may represent a promising therapeutic strategy	[93]

**Table 2:** In vivo research on integrin signaling in melanoma treatment (2021–2025).

Year	Study Model	Key Findings	Mechanism of Intervention	Reference
2025	3D mouse model/ B16F10	Inclusion of αvβ3 integrin into extracellular vesicles and subsequent transfer to recipient melanoma cells promotes migration, invasion, and metastasis	CAV1 phosphorylated on Y14 intrinsically promotes migration, invasion, and metastasis of cells	[94]
2025	3D mouse model/ B16-BL6	Carnosic acid significantly inhibits the expression of integrins: α_4_ (ITGA4) and α_9_ integrin (ITGA9)	Downregulating α_4_ (ITGA4) and α_9_ (ITGA9) integrins, carnosic acid effectively reduces the ability of melanoma cells to adhere to the extracellular matrix and proliferate	[95]

**Table 3:** In vitro and in vivo research models on integrin signaling in melanoma treatment (2021–2025).

Year	Level of Evidence	Study Model	Key Findings	Mechanism of Intervention	Reference
2025	In vitro In vivo	2D model/A2058, A375 3D mouse model/C57BL/ BALB/c	4S5NG-PE24 induced cell pyroptosis in integrin α6 melanoma cells by caspase 3/gasdermin E (GSDME) signaling pathway with lack of histological alterations	The usage of 4S5NG to deliver PE24 for selective elimination of melanoma cells through integrin α6	[49]
2024	In vitro In vivo	2D model/B16BL6 3D mouse model/B16BL6, CT26	EVs with 5-FU, DEAP-DOCA, and cRGD peptide targets αvβ3 integrin receptors on melanoma cells	EV-based drug-delivery platform capable of tumor targeting via integrin αvβ3, controlled pH-sensitive release	[96]

This section synthesizes recent research developments across six key axes that define the future of integrin-targeted melanoma therapies.

### 3.1. Peptide–Toxin Conjugates for Tumor-Selective Cytotoxicity via Integrin α6

A notable advancement in targeted melanoma therapy involves the development of the multifunctional peptide 4S5NG, which integrates an α6-specific ligand (S5), an intracellular delivery sequence (N), and an endosomal escape domain (G). When conjugated to a de-immunized Pseudomonas exotoxin (PE24), construct—4S5NG–PE24—exhibits highly selective uptake in integrin α6^+^ melanoma cells and induces pyroptotic cell death via the caspase-3/GSDME pathway.

In vivo studies confirm both selective tumor accumulation and limited off-target toxicity. Importantly, co-treatment with immune checkpoint inhibitors (anti–PD-1) leads to a synergistic anti-tumor response, supporting the dual functionality of this system in direct cytotoxicity and immune activation. This strategy establishes a promising blueprint for next-generation peptide-based therapeutics in precision oncology [49].

### 3.2. Extracellular Vesicle-Mediated Nanotherapies Targeting Integrin αvβ3 and αvβ5

Integrin-targeted extracellular vesicles (EVs) represent a versatile platform for drug delivery and intercellular communication in melanoma. EVs functionalized with the cyclic RGD peptide (cRGD) enable selective binding to αvβ3-expressing melanoma cells, while a pH-sensitive polymer (DEAP-DOCA) facilitates controlled drug release (e.g., 5-fluorouracil) in the acidic tumor microenvironment. This design enhances therapeutic selectivity and efficacy in vivo [96].

Parallel studies show that melanoma-derived ectosomes enriched with αvβ3 and αvβ5 integrins can transfer these proteins to endothelial cells, promoting angiogenesis through integrin-specific pathways. Notably, αvβ5 plays a dominant role via VEGF signaling, highlighting it as a priority target for anti-angiogenic interventions [82]. These findings confirm the utility of EVs and ectosomes in both therapy and tumor microenvironment modulation.

### 3.3. Intracellular Regulators of Integrin Expression and Chemotherapy Sensitization

Beyond extracellular targeting, emerging studies emphasize the importance of regulatory pathways modulating integrin expression and function. The SERPINB8–furin–ITGAX axis, for example, influences integrin αXβ2 expression, which contributes to melanoma cell proliferation. Targeting this axis disrupts integrin-mediated growth signals [84].

Similarly, the talin2–KANK2 module plays a pivotal role in regulating microtubule dynamics and paclitaxel sensitivity in integrin αvβ5-positive melanoma cells. Talin2 knockdown destabilizes focal adhesions and enhances chemotherapeutic response, suggesting that structural integrin interactors represent viable co-targets to improve drug efficacy [85].

Inhibition of integrin-linked kinase (ILK)—a central node in integrin signaling—combined with chloroquine, further demonstrates that translation and autophagy regulation intersect with integrin-mediated survival pathways. These regulatory insights open new ways for rational combination therapies targeting both adhesion and signaling nodes [91].

### 3.4. Integrin-Mediated Control of Angiogenesis and Metastasis

Integrins also play critical roles in tumor-induced angiogenesis and metastatic dissemination. Ectosome-mediated transfer of αvβ5 to endothelial cells promotes tube formation and migration, essential features of tumor neovascularization. Blocking αvβ5 function significantly impairs these processes, establishing it as a central effector of melanoma-associated angiogenesis [82].

On the metastatic front, carnosic acid has been shown to downregulate integrins α4 (ITGA4) and α9 (ITGA9), reducing melanoma cell adhesion and lung metastasis in vivo [95]. Meanwhile, CD36, a non-integrin membrane receptor, facilitates vasculogenic mimicry (VM) by enhancing ECM adhesion, thereby mimicking vascular channels. Together, these findings support integrin-focused anti-angiogenic and anti-metastatic strategies, especially in targeting lung dissemination and neovascular remodeling [92].

### 3.5. Integrins and Cell Fate: Senescence, Survival, and Adhesion

Recent research uncovers the roles of integrins in cellular senescence and survival, particularly through the Akt1–mTOR–p53/p21 axis. Depletion of integrin α3β1 or α2β1 in melanoma cells leads to growth arrest and senescence, accompanied by increased p53 and mTOR activity. This suggests a protective role for these integrins against premature senescence and highlights pro-senescence therapy as a potential therapeutic angle [87].

In contrast, NECTIN1, an adherens junction component, suppresses metastasis by promoting cell–cell over cell–matrix adhesion. Loss of NECTIN1 causes a phenotypic switch that facilitates matrix-dependent migration, particularly under low IGF1 conditions. These findings reveal how integrin and adhesion proteins affect melanoma cell plasticity, dissemination, and dormancy escape [89].

### 3.6. Integrins as Immunomodulators and Immunotherapy Enablers

Integrins also regulate immune responses within the tumor microenvironment. The αvβ8 integrin, expressed on regulatory T cells (Tregs), activates latent TGF-β, contributing to immune evasion. Blocking this pathway enhances anti-tumor immunity and could improve checkpoint blockade efficacy [93].

Conversely, integrin-targeted therapies can augment immune responses. The 4S5NG–PE24 conjugate, beyond inducing pyroptosis, enhances PD-1 checkpoint blockade [49], while cRGD-functionalized liposomes improve siRNA delivery against STAT3, a key immune evasion mediator [88]. These studies suggest that integrin-directed approaches can be used to recondition the immune landscape in melanoma.

The integration of integrin expression profiling into clinical diagnostics has opened new avenues for patient stratification and personalized therapy. Concentrating on particular integrin subunits may lead to early tumor detection and treatment response monitoring.

The translational research regarding integrin signaling in melanoma emphasizes its relevance to tumor biology and innovative clinical targets development. Future perspectives should elucidate heterogeneity of integrin in melanoma subtypes and combine synergistic therapeutic approaches that utilize integrin signaling vulnerabilities.

## 4. Discussion

Studies from 2021 to 2025 highlight the crucial role of integrins in melanoma progression, drug resistance, and the development of targeted therapies.

The first axis—peptide 4S5NG-PE24—selectively targets integrin α6^+^ melanoma cells, inducing pyroptosis via caspase-3/GSDME activation and enhancing anti–PD-1 immunotherapy offers tumor-specific cytotoxicity, minimal off-target toxicity, and immunogenic cell death—a rare trifecta in targeted cancer therapy. However, there are some methodological and translational limitations—like in vivo selectivity claims are based on murine models, not patient-derived xenografts (PDXs) or clinical samples—limiting predictive value for human tumors. Despite the immune-stimulatory potential of pyroptosis, clinical pyroptotic therapies remain experimental. There is a possible risk of cytokine release syndrome or immune overstimulation [49].

The extracellular vesicle-mediated nanotherapies, like melanoma-derived ectosomes, transfer αvβ3/αvβ5 integrins to endothelial cells, promoting angiogenesis by blocking αvβ5, not αvβ3, which most significantly impairs angiogenesis. This idea shifts the therapeutic focus from αvβ3 to αvβ5, challenging long-held assumptions in anti-angiogenic therapy [82]. The past literature emphasized αvβ3 as the key proangiogenic driver [97,98,99,100,101,102]. These new findings contradict prior VEGF-centric models, necessitating reassessment of cilengitide and similar compounds that primarily target αvβ3 integrin. Additionally, most studies use HUVECs and HDMECs as endothelial models, which do not fully represent tumor vasculature heterogeneity in vivo. Targeting EVs remains technically difficult—issues in delivery, tracking, and clearance are unresolved. Integrin transfer via EVs introduces off-tumor risks—altered endothelial function outside the tumor site has not been studied.

The third axis, based on targeting regulatory elements of integrin function (e.g., SERPINB8, talin2, ILK), provides a more dynamic and indirect therapeutic approach, especially for enhancing chemotherapy sensitivity and overcoming drug resistance. Some regulators (e.g., SERPINB8) have context-dependent roles: while it acts as a tumor promoter in melanoma, it functions as a suppressor in other cancers, raising concerns about off-target effects or conflicting biological roles in multi-tumor patients. ILK inhibition has systemic implications due to its pleiotropic roles in multiple cell types (cardiac, vascular, immune), and combining it with chloroquine, an autophagy inhibitor, may result in unpredictable toxicity. Chemotherapy sensitization via talin2–KANK2 or ILK pathways is largely studied in 2D monolayer cultures. These models do not replicate 3D ECM context or stromal interactions critical for integrin behavior [84,85,91]. Lack of biomarker strategies to stratify patients based on integrin regulator expression undermines the translational path for personalized medicine.

The integrin-mediated control of angiogenesis and metastasis axis confirms the role of integrins (especially αvβ5) and non-integrin adhesion receptors (e.g., CD36) in tumor angiogenesis, vasculogenic mimicry, and metastatic dissemination, particularly to the lungs. While targeting αvβ5 shows promise, blocking angiogenesis due to the tumor adaptation via alternative vascular pathways (e.g., VM) is a challenging matter [82]. CD36 inhibition could interfere with fatty acid metabolism and immune cell function, suggesting possible metabolic side effects. The anti-metastatic effects of carnosic acid, a plant-derived compound, are promising but may not be reproducible in humans due to limited bioavailability, fast metabolism, and variability in integrin expression among patient tumors. Metastatic models often use tail vein injection, which bypasses the natural steps of metastasis (e.g., intravasation and immune escape), limiting the predictive power of such findings. CD36 and integrin expression are spatiotemporally dynamic, and static expression analysis may misrepresent their actual role during metastasis [92].

The fifth axis, based on the connection of integrins and cell fate, reveals that integrins α3β1 and α2β1 are shown to prevent premature senescence, supporting a pro-survival role in melanoma [87]. Meanwhile, NECTIN1 governs the adhesion mode switch, revealing how adhesion molecules influence dormancy escape and dissemination. While promoting senescence via integrin inhibition is therapeutic in principle, senescent cells often secrete pro-inflammatory SASP factors, which can paradoxically promote tumor progression and resistance. NECTIN1 loss is associated with enhanced invasion, yet in some cancers, low NECTIN1 expression correlates with better outcomes, indicating tumor-type–specific roles that complicate generalization. Senescence is often inferred via SA β Gal activity, which lacks specificity and may not reliably indicate a stable, irreversible growth arrest. The NECTIN1 study lacks mechanistic depth on how IGF1 deficiency integrates with cell–matrix adhesion, and in vivo models (e.g., zebrafish) have limited immune context compared to human tumors [89].

Integrins as immunomodulators and immunotherapy enablers show that integrins like αvβ8 on Tregs activate TGF-β, supporting immune evasion, while integrin-targeted platforms (e.g., 4S5NG–PE24 and cRGD-siSTAT3) enhance immune checkpoint therapy and gene silencing. This axis aligns integrin biology with modern immuno-oncology paradigms. αvβ8 is not consistently expressed across melanoma subtypes, and targeting it may have limited applicability. Moreover, Tregs are essential for immune homeostasis, and systemic αvβ8 blockade could trigger autoimmune responses. Delivery platforms like cRGD liposomes face challenges in nuclease resistance, endosomal escape, and scalability for clinical use. Combining pyroptotic agents (e.g., PE24) with PD-1 inhibitors can cause excessive immune activation, raising the possibility of cytokine release syndromes or tissue damage in patients. Studies often rely on subcutaneous tumor models, which lack the complex immune architecture of human melanoma and cannot predict immune-related adverse events accurately. Absence of patient-derived melanoma models or syngeneic immunocompetent models hinders the translation of immunomodulatory findings [49,88,93].

The expanding landscape of integrin research in melanoma reflects a transition from static structural roles to dynamic regulatory and therapeutic functions. The described six axes—ranging from precision peptide–toxin delivery to immune and microenvironmental modulation—highlight integrins as multidimensional therapeutic targets.

Integrin-focused strategies are particularly promising due to their: tumor specificity (e.g., α6, αvβ3, and αvβ5), therapeutic synergy (e.g., immunotherapy and chemotherapy), delivery flexibility (e.g., EVs and liposomes), and regulatory depth (e.g., protease networks and actin-MT crosstalk). Future work should focus on enhancing clinical translatability of integrin-targeted platforms, developing isoform-specific inhibitors or ligands, exploring biomarker-guided patient stratification, and integrating multi-omic profiling to uncover novel integrin regulatory circuits.

Together, these integrin-directed innovations position themselves at the forefront of next-generation melanoma therapy.

The challenging question is whether the natural products such as vitamin D derivatives and melatonin with its metabolites [103,104], known for their anti-cancer activity [105,106,107], can sufficiently affect integrin expression and composition to attenuate melanogenesis and tumor progression, including metastatic disease. Vitamin D derivatives and melatonin with its derivatives are excellent candidates as adjuvants in melanoma therapy [1,105]. Interestingly, vitamin D hydroxymelabolites can affect fibroblast activities and composition of the ECM [108,109,110], raising the possibility that these agents could alter tumor–stroma interactions in melanoma. Their role as adjuvants in melanoma therapy warrants further investigation, particularly regarding their ability to modulate integrin composition in metastatic disease.

It is worth mentioning that acral melanoma is a distinct and rare subtype of melanoma that arises on the palms, soles, and nail beds. Unlike cutaneous melanoma, acral melanoma is not associated with ultraviolet (UV) radiation exposure, suggesting alternative mechanisms drive its initiation and progression. Recent studies indicate that integrins—transmembrane receptors involved in cell adhesion, migration, and signaling—play a pivotal role in acral melanoma biology. Altered expression of specific integrin subunits has been implicated in promoting tumor invasion, resistance to therapy, and interactions with the tumor microenvironment. Given the unique pathogenesis of acral melanoma, integrin-mediated pathways may offer subtype-specific biomarkers and therapeutic targets. Further investigation into integrin signaling could improve understanding of acral melanoma progression and guide the development of precision treatments for this challenging melanoma variant [111,112,113].

Collectively, these findings broaden the concept that the integrins are not isolated adhesion molecules, but central regulators of melanoma cell fate, invasion, immune resistance, and therapeutic responsiveness. Their subtype-specific functions, dynamic regulation, and integration into multiple oncogenic pathways make them compelling yet complex therapeutic targets. Future research should focus on integrin–ECM interactions in well-defined melanoma subtypes to evaluate combinatorial targeting approaches and explore the integration of natural compounds with established and experimental integrin-based therapies to overcome resistance and limit metastatic spread.

## 5. Conclusions

Six key research axes highlight integrins as multifaceted regulators of melanoma progression, therapeutic resistance, and immune evasion. While each axis demonstrates therapeutic potential, its translational success will depend on addressing specific contradictions, methodological gaps, and biological complexities.

Integrin-targeted therapies are no longer limited to inhibiting adhesion; they now span cytotoxic payload delivery, immune modulation, and therapy sensitization. Their integration into clinical oncology pipelines should follow a biomarker-driven, multi-modal approach, with a strong emphasis on tumor selectivity, immune context, and safety.

In conclusion, integrins have emerged as central regulators in melanoma progression, drug resistance, and immune escape, offering multiple points of therapeutic intervention. Among the current strategies, α6-targeted peptide–toxin conjugates hold clinical promise due to their tumor specificity and ability to synergize with immunotherapy. However, broader clinical success will require moving beyond isolated targets and toward integrin-guided combination therapies that integrate immune modulation, metastasis inhibition, and drug delivery. Future research must prioritize biomarker-driven patient stratification, validation in complex, immunocompetent models, and the development of real-time imaging tools to monitor integrin activity. Clinically, integrins should no longer be viewed as passive adhesion molecules but as actionable hubs for precision oncology.

## Data Availability

Not applicable.

## Abbreviations

The following abbreviations are used in this manuscript:

AKT	Protein Kinase B
AMPK	AMP-Activated Protein Kinase
BRAF	B-type Rapidly Accelerated Fibrosarcoma
CD11c	Cluster of Differentiation 11c
CD36	Cluster of Differentiation 36
CD44	Cluster of Differentiation 44
c-RGD	Cyclic RGD
CRISPR	Clustered Regularly Interspaced Short Palindromic Repeats technique
CQ	Chloroquine
CYR61	Cysteine-Rich Angiogenic Inducer 61
DDR	Discoidin Domain Receptor 1/2
DEAP DOCA	Diethylaminoethyl-Polymer and Deoxycholic Acid
ECM	Extracellular Matrix
EGF	Epidermal Growth Factor
EMT	Epithelial–Mesenchymal Transition
ERK	Extracellular Signal-Regulated Kinase (1/2)
EVs	Extracellular Vesicles
FAK	Focal Adhesion Kinase
GSDME	Gasdermin E
GGPP	Geranylgeranyl Pyrophosphate
GTP	Guanosine Triphosphate
HDMECs	Human Dermal Microvascular Endothelial Cells
HMGCR	3-Hydroxy-3-Methylglutaryl-Coenzyme A Reductase
HUVECs	Human Umbilical Vein Endothelial Cells
IGF1	Insulin-like Growth Factor 1
ILK	Integrin-Linked Kinase
ITGA1	Integrin Alpha-1
ITGAX	Integrin Alpha-X (CD11c)
Itgβ8	Integrin Beta-8
JNK	C-Jun N-terminal Kinase
KANK2	KN motif and Ankyrin Repeat Domain-Containing Protein 2
MAPK	Mitogen-Activated Protein Kinase
MEK	Mitogen-Activated Protein Kinase (1/2)
MLC	Myosin Light Chain
MYPT1	Myosin Phosphatase Target Subunit 1
NECTIN1	Nectin Cell Adhesion Molecule 1
RGD	Arginine-Glycine-Aspartic Acid Peptide
SA-β-Gal	Senescence-Associated Beta-Galactosidase
SERPINB8	Serine Protease Inhibitor B8
siILK	Small Interfering RNA Targeting Integrin-Linked Kinase
siRNA	Small Interfering Ribonucleic Acid
STAT3	Signal Transducer and Activator of Transcription 3
TGF-β	Transforming Growth Factor Beta
mTOR/C1	Mechanistic Target of Rapamycin (Complex 1)
MT	Microtubules
P	Phosphorylation
p130Cas	Crk-Associated Substrate
PAC/PTX	Paclitaxel
PGC1α	Peroxisome Proliferator-Activated Receptor Gamma Coactivator 1-Alpha
PI3K	Phosphoinositide 3-Kinase
PD-1	Programmed Cell Death Protein 1
PE24	De-immunized Exotoxin A
RAF	Rapidly Accelerated Fibrosarcoma
RAS	Active State Ras Protein
RhoA-Ras	Homolog Family Member A
ROS	Reactive Oxygen Species
RTK	Receptor Tyrosine Kinase
SA β Gal	Senescence-associated β-Galactosidase
SASP	Senescence-associated Secretory Phenotype
SRC	Proto-Oncogene Tyrosine-Protein Kinase
TAZ	Transcriptional coactivator
Treg	Regulatory T Cell
VM	Vasculogenic Mimicry
YAP	Yes-Associated Protein,
5-FU	5-Fluorouracil

## References

[1] Slominski R.M., Kim T.K., Janjetovic Z., Brozyna A.A., Podgorska E., Dixon K.M., Mason R.S., Tuckey R.C., Sharma R., Crossman D.K. (2024). Malignant Melanoma: An Overview, New Perspectives, and Vitamin D Signaling. Cancers.

[2] Reinhart J., Campbell E., Proffer S., Crum O., Todd A., Gibson L., Brewer J., Demer A. (2024). Incidence and mortality trends of primary cutaneous melanoma: A 50-year Rochester Epidemiologic Project study. JAAD Int..

[3] Dennis L. (2022). Cumulative Sun Exposure and Melanoma in a Population-Based Case–Control Study: Does Sun Sensitivity Matter?. Cancers.

[4] Le Clair M., Cockburn M. (2016). Tanning bed use and melanoma: Establishing risk and improving prevention interventions. Prev. Med. Rep..

[5] Slominski R.M., Chen J.Y., Raman C., Slominski A.T. (2024). Photo-neuro-immuno-endocrinology: How the ultraviolet radiation regulates the body, brain, and immune system. Proc. Natl. Acad. Sci. USA.

[6] Slominski A.T., Zmijewski M.A., Plonka P.M., Szaflarski J.P., Paus R. (2018). How UV Light Touches the Brain and Endocrine System Through Skin, and Why. Endocrinology.

[7] Slominski R.M., Raman C., Jetten A.M., Slominski A.T. (2025). Neuro-immuno-endocrinology of the skin: How environment regulates body homeostasis. Nat. Rev. Endocrinol..

[8] Haussmann P., Pavani C., Marcolongo-Pereira C., Bellettini-Santos T., da Silva B., Benedito I., Freitas M., Baptista M., Chiarelli-Neto O. (2022). Melanin photosensitization by green light reduces melanoma tumor size. J. Photochem. Photobiol..

[9] Slominski A., Tobin D.J., Shibahara S., Wortsman J. (2004). Melanin pigmentation in mammalian skin and its hormonal regulation. Physiol. Rev..

[10] Slominski R.M., Sarna T., Płonka P.M., Raman C., Brożyna A.A., Slominski A.T. (2022). Melanoma, Melanin, and Melanogenesis: The Yin and Yang Relationship. Front. Oncol..

[11] Jackson A., Wilkinson C., Pill R. (1999). Moles and melanomas--who’s at risk, who knows, and who cares? A strategy to inform those at high risk. Br. J. Gen. Pract..

[12] Funchain P., Ni Y., Heald B., Bungo B., Arbesman M., Behera T.R., McCormick S., Song J., Kennedy L., Nielsen S. (2024). Germline cancer susceptibility in individuals with melanoma. J. Am. Acad. Dermatol..

[13] Lynch H., Brand R., Hogg D., Deters C., Fusaro R., Lynch J., Liu L., Knezetic J., Lassam N., Goggins M. (2002). Phenotypic variation in eight extended CDKN2A germline mutation familial atypical multiple mole melanoma–pancreatic carcinoma–prone families. Cancer.

[14] Robbins H., Clarke C., Arron S., Tatalovich Z., Kahn A., Hernandez B., Paddock L., Yanik E., Lynch C., Kasiske B. (2015). Melanoma Risk and Survival among Organ Transplant Recipients. J. Investig. Dermatol..

[15] Rutkowski P., Wysocki P.J., Nasierowska-Guttmejer A., Jeziorski A., Wysocki W., Kalinka E., Świtaj T., Kozak K., Kamińska-Winciorek G., Czarnecka A. (2020). Cutaneous Melanoma. Oncol. Clin. Pract..

[16] Damsky W.E., Rosenbaum L.E., Bosenberg M. (2011). Decoding Melanoma Metastasis. Cancers.

[17] Younes R., Abrao F.C., Gross J. (2013). Pulmonary metastasectomy for malignant melanoma. Melanoma Res..

[18] Guruvaiah S., Guruvaiah S.N., Li W., Ponnatapura J. (2023). A rare radiological presentation of pulmonary metastases from malignant melanoma. Radiol. Case Rep..

[19] Pedersen S., Johansen E., Højholt K., Pedersen M., Mogensen A., Petersen S., Haslund C., Donia M., Schmidt H., Basdtholt L. (2025). Survival improvements in patients with melanoma brain metastases and leptomeningeal disease in the modern era: Insights from a nationwide study (2015–2022). Eur. J. Cancer.

[20] So J.-K., Hong J.-Y., Chung M.-W., Cho S.-B. (2021). A Case of Metastatic Melanoma in the Liver Mimicking Hepatocellular Carcinoma. J. Liver Cancer.

[21] Slominski R.M., Raman C., Chen J.Y., Slominski A.T. (2023). How cancer hijacks the body’s homeostasis through the neuroendocrine system. Trends Neurosci..

[22] Livingstone E., Zaremba A., Horn S., Ugurel S., Casalini B., Schlaak M., Hassel J., Herbst R., Utikal J., Weide B. (2020). GNAQ and GNA11 mutant nonuveal melanoma: A subtype distinct from both cutaneous and uveal melanoma. Br. J. Dermatol..

[23] Gurung S., Gurung T., Mallela K., Jenkins B., von Kriegsheim A., Manrique E., Millan-Esteban D., Romero-Camarero I., Amaral F., Craig S. (2025). Stromal lipid species dictate melanoma metastasis and tropism. Cancer Cell.

[24] Slominski A., Kim T.K., Brozyna A.A., Janjetovic Z., Brooks D.L., Schwab L.P., Skobowiat C., Jozwicki W., Seagroves T.N. (2014). The role of melanogenesis in regulation of melanoma behavior: Melanogenesis leads to stimulation of HIF-1alpha expression and HIF-dependent attendant pathways. Arch. Biochem. Biophys..

[25] Slominski A., Zmijewski M.A., Pawelek J. (2012). L-tyrosine and L-dihydroxyphenylalanine as hormone-like regulators of melanocyte functions. Pigment. Cell Melanoma Res..

[26] Slominski A., Zbytek B., Slominski R. (2009). Inhibitors of melanogenesis increase toxicity of cyclophosphamide and lymphocytes against melanoma cells. Int. J. Cancer.

[27] Slominski A.T., Carlson J.A. (2014). Melanoma resistance: A bright future for academicians and a challenge for patient advocates. Mayo Clin. Proc..

[28] Piskounova E., Agathocleous M., Murphy M., Hu Z., Huddlestun S.E., Zhao Z., Leitch M., Johnson T., Deberardinis R., Morrison S. (2015). Oxidative stress inhibits distant metastasis by human melanoma cells. Nature.

[29] Gargett T., Abbas M., Rolan P., Price J., Gosling K.M., Ferrante A., Ruszkiewicz A., Atmosukarto I., Altin j., Parish C. (2018). Phase I trial of Lipovaxin-MM, a novel dendritic cell-targeted liposomal vaccine for malignant melanoma. Cancer Immunol. Immunother..

[30] Karimkhani C., Green A., Nijsten T., Weinstock M., Dellavalle R., Naghavi M., Fitzmaurice C. (2017). The global burden of melanoma: Results from the Global Burden of Disease Study 2015. Br. J. Dermatol..

[31] Siegel R., Miller K., Fuchs H., Jemal A. (2022). Cancer statistics, 2022. CA Cancer J. Clin..

[32] Ramirez N., Zhang Z., Madamanchi A., Boyd K., O’Rear L., Nashabi A., Li Z., Dupont W., Zijlstra A., Zutter M. (2022). The α_2_β_1_ integrin is a metastasis suppressor in mouse models and human cancer. J. Clin. Investig..

[33] Xiong J., Stehle T., Diefenbach B., Zhang R., Dunker R., Scott D.L., Joachimiak A., Goodman S., Arnaout M. (2001). Crystal structure of the extracellular segment of integrin alpha Vbeta3. Science.

[34] Okada T., Lee A., Qin Y., Li-Xuan A., Narasimhan M., Takahiro S., Shen Y., O’Connor R., Lopez-Lago M., Craig A. (2016). Integrin-α10 Dependency Identifies RAC and ICTOR as Therapeutic Targets in High-Grade Myxofibrosarcoma. Cancer Discov..

[35] Almeida E., Ilić D., Han Q., Hauck C., Jin F., Kawakatsu H., Schlaepfer D., Damsky C. (2000). Matrix Survival Signaling. J. Cell Biol..

[36] Anderson J., Li J., Springer T. (2022). Regulation of integrin α5β1 conformational states and intrinsic affinities by metal ions and the ADMIDAS. Mol. Biol. Cell.

[37] Wegener K., Partridge A., Han J., Pickford A., Liddington R., Ginsberg M., Campbell I. (2007). Structural Basis of Integrin Activation by Talin. Cell.

[38] de Fougerolles A., Sprague A., Nickerson-Nutter C.h., Chi-Rosso G., Rennert P., Gardner H., Gotwals P., Lobb R., Koteliansky V. (2000). Regulation of inflammation by collagen-binding integrins α1β1 and α2β1 in models of hypersensitivity and arthritis. J. Clin. Investig..

[39] Arimori T., Miyazaki N., Mihara E., Takizawa M., Taniguchi Y., Cabañas C., Sekiguchi K., Takagi J. (2021). Structural mechanism of laminin recognition by integrin. Nat. Commun..

[40] Lahti M., Heino J., Käpylä J. (2013). Leukocyte integrins αLβ2, αMβ2 and αXβ2 as collagen receptors—Receptor activation and recognition of GFOGER motif. Int. J. Biochem. Cell Biol..

[41] Mauri E., Sacchetti A., Vicario N., Peruzzotti-Jametti L., Rossi F., Pluchino S. (2018). Evaluation of RGD functionalization in hybrid hydrogels as 3D neural stem cell culture systems. Biomater. Sci..

[42] Li J., Ma J., Zhang Q., Gong H., Gao D., Wang Y., Li B., Li X., Zheng H., Wu Z. (2022). Spatially resolved proteomic map shows that extracellular matrix regulates epidermal growth. Nat. Commun..

[43] Dyring-Andersen B., Løvendorf M., Coscia F., Santos A., Møller L., Colaço A. (2020). Spatially and cell-type resolved quantitative proteomic atlas of healthy human skin. Nat. Commun..

[44] Kaur A., Ecker B., Douglass S., Kugel C., Webster M., Almeida F., Somasundaram R., Hayden J., Ban E., Ahmadzadech H. (2019). Remodeling of the Collagen Matrix in Aging Skin Promotes Melanoma Metastasis and Affects Immune Cell Motility. Cancer Discov..

[45] Chen C., Mrksich M., Huang S., Whitesides G., Ingber D. (1997). Geometric control of cell life and death. Science.

[46] Hynes R. (2004). The emergence of integrins: A personal and historical perspective. Matrix Biol..

[47] Levi L., Toyooka T., Patarroyo M., Frisan T. (2015). Bacterial genotoxins promote inside-out integrin β1 activation, formation of focal adhesion complexes and cell spreading. PLoS ONE.

[48] Nicolas-Boluda A., Vaquero J., Vimeux L., Guilbert T., Barrin S., Kantari-Mimoun C., Ponzo M., Renault G., Deptula P., Pogoda K. (2021). Tumor stiffening reversion through collagen crosslinking inhibition improves T cell migration and anti-PD-1 treatment. Elife.

[49] Zhao Z., Li Z.-Q., Huang Y.-B., Liu M., Cao F., Bu G.-L., Xu P.-F., Fang Q., Hu Z.-L., Wu D. (2025). An optimized integrin α6-targeted peptide capable of delivering toxins for melanoma treatment. J. Transl. Med..

[50] Alanko J., Mai A., Jacquemet G., Schauer K., Kaukonen R., Saari M. (2015). Integrin endosomal signalling suppresses anoikis. Nat. Cell Biol..

[51] Alabi S., Jaime-Figueroa S., Yao Z., Gao Y., Hines J., Samarasinghe K., Vogt L., Rosen N., Crews C. (2021). Mutant-selective degradation by BRAF-targeting PROTACs. Nat. Commun..

[52] Yu C., Zhang M., Song J., Zheng X., Xu G., Bao Y., Lan J., Luo D., Hu J., Li J. (2020). Integrin-Src-YAP1 signaling mediates the melanoma acquired resistance to MAPK and PI3K/mTOR dual targeted therapy. Mol. Biomed..

[53] Mitchell K., Svenson K.B., Longmate W., Gkirtzimanaki K., Sadej R., Wang X., Zhao J., Eliopoulos A., Berdithevski F., Dipersio C. (2010). Suppression of integrin α_3_β_1_ in breast cancer cells reduces COX-2 gene expression and inhibits tumorigenesis, invasion, and crosstalk to endothelial cells. Cancer Res..

[54] Albelda S., Mette S., Elder D., Stewart R., Damjanovich L., Herlyn, Buck C. (1990). Integrin distribution in malignant melanoma: Association of the β3 subunit with tumor progression. Cancer Res..

[55] Wong R., Ng P., Dedhar S., Li G. (2007). The role of integrin-linked kinase in melanoma cell migration, invasion, and tumor growth. Mol. Cancer Ther..

[56] Watson-Hurst K., Becker D. (2006). The role of N-Cadherin, MCAM, and β3 integrin in melanoma progression, proliferation, migration and invasion. Cancer Biol. Ther..

[57] Lydolph M., Morgan-Fisher M., Høye A., Couchman J., Wewer, Yoneda A. (2009). α9β1 integrin in melanoma cells can signal different adhesion states for migration and anchorage. Exp. Cell Res..

[58] Voura E., Ramjeesingh R., Montgomery A., Siu C. (2001). Involvement of integrin αvβ3and cell adhesion molecule l1 in transendothelial migration of melanoma cells. Mol. Biol. Cell.

[59] Bosserhoff A., Stoll R., Sleeman J., Bataille F., Buettner R., Holak T. (2003). Active detachment involves inhibition of cell-matrix contacts of malignant melanoma cells by secretion of melanoma inhibitory activity. Lab. Investig..

[60] Anggradita L., Kim J., Kim M., Son J., Farhan M., Jeberson J. (2025). Targeting the integrin beta 1-focal adhesion kinase axis with artemisinin: Biophysical disruption of cell adhesion, migration, and invasion in tongue cancer. View.

[61] McKenzie J., Liu T., Goodson A., Grossman D. (2010). Survivin enhances motility of melanoma cells by supporting Akt activation and α5 integrin upregulation. Cancer Res..

[62] Mitjans F., Meyer T., Fittschen C., Goodman S., Jonczyk A., Marshall J. (2000). In vivo therapy of malignant melanoma by means of antagonists of αv integrins. Int. J. Cancer.

[63] Desch A., Strozyk E., Bauer A., Huck V., Niemeyer V., Wieland T. (2012). Highly invasive melanoma cells activate the vascular endothelium via an MMP-2/Integrin αvβ5–induced secretion of VEGF-A. Am. J. Patol..

[64] Hegerfeldt Y., Tusch M., Bröcker E., Friedl P. (2002). Collective cell movement in primary melanoma explants: Plasticity of cell-cell interaction, β1-integrin function, and migration strategies. Cancer Res..

[65] Leonard M., Novak M., Snyder D., Snow G., Pamidimukkala N., McCorkle J. (2019). The metastasis suppressor NME1 inhibits melanoma cell motility via direct transcriptional induction of the integrin beta-3 gene. Exp. Cell Res..

[66] Vannini A., Leoni V., Barboni C., Sanapo M., Zaghini A., Malatesta P. (2019). αvβ3-integrin regulates PD-L1 expression and is involved in cancer immune evasion. Proc. Natl. Acad. Sci. USA.

[67] Quattrocchi E., Sominidi-Damodaran S., Murphree D., Meves A. (2020). β3 integrin immunohistochemistry as a method to predict sentinel lymph node status in patients with primary cutaneous melanoma. Int. J. Dermatol..

[68] Nurzat Y., Su W., Min P., Li K., Xu H., Zhang Y. (2021). Identification of therapeutic targets and prognostic biomarkers among integrin subunits in the skin cutaneous melanoma microenvironment. Front. Oncol..

[69] Militaru I., Rus A., Munteanu C., Manica G., Petrescu S. (2023). New panel of biomarkers to discriminate between amelanotic and melanotic metastatic melanoma. Front. Oncol..

[70] Xie R., Li B., Jia L., Li Y. (2022). Identification of core genes and pathways in melanoma metastasis via bioinformatics analysis. Int. J. Mol. Sci..

[71] Zeltz C., Navab R., Heljasvaara R., Kusche-Gullberg M., Lu N., Tsao M., Gullberg D. (2022). Integrin α_11_β_1_ in tumor fibrosis: More than just another cancer-associated fibroblast biomarker?. J. Cell Commun. Signal..

[72] Talluri B., Amar K., Saul M., Shireen T., Konjufca V., Ma J., Ha T., Chowdhury F. (2020). COL2A1 is a novel biomarker of melanoma tumor repopulating cells. Biomedicines.

[73] Rezaie Y., Fattahi F., Mashinchi B., Kamyab Hesari K., Montazeri S., Kalantari, Madjd Z., Zanjani L. (2023). High expression of Talin-1 is associated with tumor progression and recurrence in melanoma skin cancer patients. BMC Cancer.

[74] Shidal C., Singh N., Nagarkatti P., Nagarkatti M. (2019). MicroRNA-92 expression in CD133+ melanoma stem cells regulates immunosuppression in the tumor microenvironment via integrin-dependent activation of TGFβ. Cancer Res..

[75] Cui J., Shu C., Xu J., Chen D., Li J., Ding K. (2020). JP1 suppresses proliferation and metastasis of melanoma through MEK1/2 mediated NEDD4L-SP1-Integrin αvβ3 signaling. Theranostics.

[76] Ghafouri-Fard S., Gholipour M., Taheri M. (2021). MicroRNA signature in melanoma: Biomarkers and therapeutic targets. Front. Oncol..

[77] Surman M., Kędracka-Krok S., Hoja-Łukowicz D., Jankowska U., Drożdż A., Stępień E. (2020). Mass spectrometry-based proteomic characterization of cutaneous melanoma ectosomes reveals the presence of cancer-related molecules. Int. J. Mol. Sci..

[78] Zhou Y., Ge M., Li X., Xie Q., Sun M., Li K. (2025). Synthesis and Preclinical Evaluation of Dual-Targeted Molecular Tracer for Positron Emission Tomography Imaging of Programmed Cell Death Ligand-1 and Integrin α_v_β_3_. ACS Pharmacol. Transl. Sci..

[79] Nguyen A., Heim J., Cordara G., Chan M., Johannesen H., Charlesworth C., Li M., Azumaya C., Madden B., Krengel U. (2025). Structural and functional characterization of integrin α5-targeting antibodies for anti-angiogenic therapy. bioRxiv.

[80] Pozniak J., Roda N., Landeloos E., Antoranz A., Van Herck Y., De Visscher A., Demaerel P., Vanwynsberghe L., Declercq J., Gkemisis C. (2025). Cytotoxic NK Cells Impede Response to Checkpoint Immunotherapy in Melanoma with an Immune-Excluded Phenotype. Cancer Discov..

[81] Słaby J., Wnuk M., Błoniarz D., Stec P., Szmatoła T., Kaznowska E., Reich A., Moros M., Lewinska A. (2025). ITGA1, the alpha 1 subunit of integrin receptor, is a novel marker of drug-resistant senescent melanoma cells in vitro. Arch. Toxicol..

[82] Surman M., Wilczak M., Bzowska M., Tylko G., Przybyło M. (2024). The Proangiogenic Effects of Melanoma-Derived Ectosomes Are Mediated by αvβ5 Integrin Rather than αvβ3 Integrin. Cells.

[83] Lee Y., Kim K., Park S., Kim D., Choi Y., Nam H., Lee S., Cho M. (2024). CYR61 Is Overexpressed in Human Melanoma Tissue Ex Vivo and Promotes Melanoma Cell Survival and Proliferation Through Its Binding Ligand Integrin β3 In Vitro. Ann. Dermatol..

[84] Ni L., Li P., Li M., Huang S., Dang N. (2023). SERPINB8 and furin regulate ITGAX expression and affect the proliferation and invasion of melanoma cells. Exp. Dermatol..

[85] Lončarić M., Stojanović N., Rac-Justament A., Coopmans K., Majhen D., Humphries J. (2023). Talin2 and KANK2 functionally interact to regulate microtubule dynamics, paclitaxel sensitivity and cell migration in the MDA-MB-435S melanoma cell line. Cell. Mol. Biol. Lett..

[86] Liang J., Yu D., Luo C., Bennet C., Jedrychowski M., Gygi S., Widlund H., Puigserver P. (2023). Epigenetic suppression of PGC1α (PPARGC1A) causes collateral sensitivity to HMGCR-inhibitors within BRAF-treatment resistant melanomas. Nat. Commun..

[87] Morozevich G., Kozlova N., Gevorkian N., Berman A. (2022). The Integrin α3β1 Signaling in the Regulation of the SK-Mel-147 Melanoma Cell Senescence. Biochem. Mosc. Suppl. B Biomed. Chem..

[88] Khabazian E., Vakhshiteh F., Norouzi P., Fatahi Y., Dinarvand R., Atyabi F. (2022). Cationic liposome decorated with cyclic RGD peptide for targeted delivery of anti-STAT3 siRNA to melanoma cancer cells. J. Drug Target..

[89] Ablain J., Al Mahi A., Rothschild H., Prasad M., Aires S., Yang S., Dokukin M., Xu S., Dang M., Sokolov I. (2022). Loss of NECTIN1 triggers melanoma dissemination upon local IGF1 depletion. Nat. Genet..

[90] Kozlova N., Morozevich G., Berman A. (2021). Implication of integrin α2β1 in senescence of SK-Mel-147 human melanoma cells. Aging.

[91] Gil D., Laidler P., Zarzycka M., Dulińska-Litewka J. (2021). Inhibition effect of chloroquine and integrin-linked kinase knockdown on translation in melanoma cells. Int. J. Mol. Sci..

[92] Martini C., DeNichilo M., King D., Cockshell M., Ebert B., Dale B. (2021). CD36 promotes vasculogenic mimicry in melanoma by mediating adhesion to the extracellular matrix. BMC Cancer.

[93] Lainé A., Labiad O., Hernandez-Vargas H., This S., Sanlaville A., Léon S. (2021). Regulatory T cells promote cancer immune-escape through integrin αvβ8-mediated TGF-β activation. Nat. Commun..

[94] Huilcaman R., Campos A., Contreras P., Simón M., Varas-Godoy F., Grünenwald B.-S., Shao B., Heinecke J., Loboz-Gonzalez L., Leyton L. (2025). Quest. Inclusion of αvβ3 integrin into extracellular vesicles in a caveolin-1 tyrosine-14-phosphorylation dependent manner and subsequent transfer to recipient melanoma cells promotes migration, invasion and metastasis. Cell Commun. Signal.

[95] Shibata S., Yamada K., Shigeyuki K. (2025). Carnosic acid inhibits integrin expression and prevents pulmonary metastasis of melanoma. Biosci. Biotechnol. Biochem..

[96] Kim J., Lee E., Lee E. (2024). Development of 5-Fluorouracil/pH-Responsive Adjuvant-Embedded Extracellular Vesicles for Targeting α_v_β_3_ Integrin Receptors in Tumors. Pharmaceutics.

[97] Yuan R., Li Y., Yang B., Jin Z., Xu J., Shao Z., Miao H., Ren T., Yang Y., Li G. (2021). LOXL1 exerts oncogenesis and stimulates angiogenesis through the LOXL1-FBLN5/αvβ3 integrin/FAK-MAPK axis in ICC. Mol. Ther. Nucl. Acids.

[98] Nakagawa T., Ohta K., Naruse T., Sakuma M., Fukada S., Yamakado N., Akagi M., Sasaki K., Niwata C., Ono S. (2022). Inhibition of angiogenesis and tumor progression of MK-0429, an integrin αvβ3 antagonist, on oral squamous cell carcinoma. J. Cancer Res. Clin. Oncol..

[99] Li J., Fukase Y., Shang Y., Zou W., Muñoz-Félix J., Buitrago L., van Aghhoven J., Zhang Y., Hara R., Tanaka Y. (2019). Novel pure αvβ3 integrin antagonists that do not induce receptor extension, prime the receptor, or enhance angiogenesis at low concentrations. ACS Pharmacol. Transl. Sci..

[100] Lin X., Sun Y., Yang S., Yu M., Pan L., Yang J., Yang j., Shao Q., Liu J., Liu Y. (2021). Omentin-1 modulates macrophage function via integrin receptors αvβ3 and αvβ5 and reverses plaque vulnerability in animal models of atherosclerosis. Front. Cardiovasc. Med..

[101] Zeng L., Nie H., Xiong H., Yang Z., Hu X., Su T. (2025). A Study on Regulating OSCC Cell Function by Blocking Integrin αvβ3 With Cilengitide. J. Oral Patol. Med..

[102] Ludwig B., Krautkremer N., Tomassi S., Di Maro S., Di Leva F., Benge A., Nieberler M., Kessler H., Marinelli L., Kossatz S. (2025). Switching Roles—Exploring Concentration-Dependent Agonistic versus Antagonistic Behavior of Integrin Ligands. J. Med. Chem..

[103] Kim T.K., Slominski R.M., Pyza E., Kleszczynski K., Tuckey R.C., Reiter R.J., Holick M.F., Slominski A.T. (2024). Evolutionary formation of melatonin and vitamin D in early life forms: Insects take centre stage. Biol. Rev. Camb. Philos. Soc..

[104] Adamiak K., Gaida V.A., Schäfer J., Bosse L., Diemer C., Reiter R.J., Slominski A.T., Steinbrink K., Sionkowska A., Kleszczyński K. (2024). Melatonin/Sericin Wound Healing Patches: Implications for Melanoma Therapy. Int. J. Mol. Sci..

[105] Slominski A.T., Kim T.K., Janjetovic Z., Slominski R.M., Ganguli-Indra G., Athar M., Indra A.K., Reiter R.J., Kleszczynski K. (2025). Melatonin and the Skin: Current Progress and Perspectives for Human Health. J. Investig. Dermatol..

[106] Slominski A.T., Kim T.K., Janjetovic Z., Slominski R.M., Li W., Jetten A.M., Indra A.K., Mason R.S., Tuckey R.C. (2024). Biological Effects of CYP11A1-Derived Vitamin D and Lumisterol Metabolites in the Skin. J. Investig. Dermatol..

[107] Reiter R.J., De Almeida Chuffa L.G., Simão V.A., Martín Giménez V.M., De Las Heras N., Spandidos D.A., Manucha W. (2024). Melatonin and vitamin D as potential synergistic adjuvants for cancer therapy (Review). Int. J. Oncol..

[108] Janjetovic Z., Qayyum S., Reddy S.B., Podgorska E., Scott S.G., Szpotan J., Mobley A.A., Li W., Boda V.K., Ravichandran S. (2024). Novel Vitamin D3 Hydroxymetabolites Require Involvement of the Vitamin D Receptor or Retinoic Acid-Related Orphan Receptors for Their Antifibrogenic Activities in Human Fibroblasts. Cells.

[109] Janjetovic Z., Postlethwaite A., Kang H.S., Kim T.K., Tuckey R.C., Crossman D.K., Qayyum S., Jetten A.M., Slominski A.T. (2021). Antifibrogenic Activities of CYP11A1-derived Vitamin D3-hydroxyderivatives Are Dependent on RORgamma. Endocrinology.

[110] Brown Lobbins M.L., Scott I.O., Slominski A.T., Hasty K.A., Zhang S., Miller D.D., Li W., Kim T.K., Janjetovic Z., Patel T.S. (2021). 17,20S(OH)_2_pD Can Prevent the Development of Skin Fibrosis in the Bleomycin-Induced Scleroderma Mouse Model. Int. J. Mol. Sci..

[111] Yin M., Zhang Y., Wang W., Zhao S., Su J., Li S., Chen X. (2025). Identification of two molecularly and prognostically distinct subtypes in acral melanoma using network prediction method. J. Eur. Acad. Dermatol. Venereol..

[112] Farshidfar F., Rhrissorrakrai K., Levovitz C., Peng C., Knight J., Bacchiocchi A., Su H., Yin M., Sznol M., Ariyan S. (2022). Integrative molecular and clinical profiling of acral melanoma links focal amplification of 22q11. 21 to metastasis. Nat. Commun..

[113] Farshidfar F., Peng C., Levovitz C., Knight J., Bacchiocchi A., Su J., Rhrissorrakrai K., Yin M., Sznol M., Ariyan S. (2021). Integrative molecular and clinical profiling of acral melanoma identifies LZTR1 as a key tumor promoter and therapeutic target. bioRxiv.

